# Pharmacoinformatic Investigation of Medicinal Plants from East Africa

**DOI:** 10.1002/minf.202000163

**Published:** 2020-10-08

**Authors:** Conrad V. Simoben, Ammar Qaseem, Aurélien F. A. Moumbock, Kiran K. Telukunta, Stefan Günther, Wolfgang Sippl, Fidele Ntie‐Kang

**Affiliations:** ^1^ Institute of Pharmacy Martin-Luther University of Halle-Wittenberg Kurt-Mothes-Str. 3 06120 Halle/Saale Germany; ^2^ Institute of Pharmaceutical Sciences, Research Group Pharmaceutical Bioinformatics Albert-Ludwigs-University Freiburg Hermann-Herder-Straße 9 79104 Freiburg Germany; ^3^ ELIXIR@PSB, VIB-UGent Center for Plant Systems Biology Technologiepark 71 9052 Ghent Belgium; ^4^ Department of Chemistry, Faculty of Science University of Buea P.O. Box 63 Buea CM 00237 Cameroon; ^5^ Institut für Botanik Technische Universität Dresden ZellescherWeg 20b 01217 Dresden Germany

**Keywords:** database, drug discovery, Eastern Africa, medicinal plants, natural products (NPs)

## Abstract

Medicinal plants have widely been used in the traditional treatment of ailments and have been proven effective. Their contribution still holds an important place in modern drug discovery due to their chemical, and biological diversities. However, the poor documentation of traditional medicine, in developing African countries for instance, can lead to the loss of knowledge related to such practices. In this study, we present the Eastern Africa Natural Products Database (EANPDB) containing the structural and bioactivity information of 1870 unique molecules isolated from about 300 source species from the Eastern African region. This represents the largest collection of natural products (NPs) from this geographical region, covering literature data of the period from 1962 to 2019. The computed physicochemical properties and toxicity profiles of each compound have been included. A comparative analysis of some physico‐chemical properties like molecular weight, H‐bond donor/acceptor, logP_o/w_, etc. as well scaffold diversity analysis has been carried out with other published NP databases. EANPDB was combined with the previously published Northern African Natural Products Database (NANPDB), to form a merger African Natural Products Database (ANPDB), containing ∼6500 unique molecules isolated from about 1000 source species (freely available at http://african‐compounds.org). As a case study, latrunculins A and B isolated from the sponge *Negombata magnifica* (Podospongiidae) with previously reported antitumour activities, were identified via substructure searching as molecules to be explored as putative binders of histone deacetylases (HDACs).

## Graphical abstract

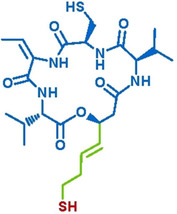

## 1 Introduction

Historically, natural products (NPs), i. e. compounds derived from natural sources (bacterial, fungi, plants or animal species) possessing biological activities; have been the primary provenance of medicine globally.^[1]^ Although the approval rate of new drugs from nature has not increased proportionally with the financial and technological investments on NP researches,^[2]^ NPs still account for about half of the FDA‐approved drugs..[^2a^, ^2d^, ^3^] Thus, seeing the remarkable contribution of NPs as drugs, huge amounts of NPs are being isolated and characterized daily. Also, the biological evaluations of the isolated molecules are carried out in order to confirm the therapeutic claims. Further studies on the establishment of the mechanisms of actions of the isolated biologically interesting NPs are being carried out with the hope of getting the next generation lead compounds for drug discovery.^[4]^

One of the magnificent beauties of the African continent is its richness in flora and fauna. This richness offers the African population diverse traditional means in treating ailments based on what nature has presented to them. However, due to poor documentation, some of this traditional information is being lost nowadays. This is one of the main factors behind the scientific exploration of the known traditional methods as well as the source species (bacteria, fungi, plants or animals). Accordingly, several NPs have been identified and collected into several public and commercial databases and repositories.[^4g^, ^5^, ^6a^] However, analysis, e. g. by using principal component analysis (PCA) of the available NP datasets show that only a small portion of the already annotated NPs originate from Africa or even occupying similar chemical space to the current collection of African NPs.^[6]^

Many of the medicinal plants from the Eastern African region have illustrated interesting values in traditional medicine, which makes use of indigenous knowledge to treat diseases.^[7]^ This paper describes a collection of 1870 NPs from 302 species belonging to 58 families (some having usage in traditional medicine), with data coverage from 1962 to 2019, as well as a pharmacoinformatic analysis of the compound data. This novel electronic dataset, called East African Natural Products Database (EANPDB) provides interesting information regarding the original literature sources and currently stands out as the largest collection of NPs from Eastern Africa. The molecular structures and metadata of EANPDB are accessible as supplementary files to this article. Furthermore, EANPDB was combined to the previously published Northern African Natural Products Database (NANPDB),^[6c]^ to form a merger called the African Natural Products Database (ANPDB), which is freely available at http://african‐compounds.org. The combined dataset resulted in ∼6500 unique molecules.

## 2 Methods

### 2.1 Data Curation

The data was collected as part of our ongoing project, which is the development of chemical libraries of natural products from African medicinal plants, marine, fungal and bacterial sources.[^6b^, ^6c^, ^6e^, ^6f^] The unified ANPDB is constantly being updated based on inputs from journal articles and MSc/PhD. thesis from African university libraries of various regions. In the current study, emphasis was laid on data published on compounds identified from source species harvested in the Eastern African region (including the countries; Burundi, Ethiopia, Kenya, Rwanda, Tanzania, Uganda). The data for the source species, compounds, literature references and the use of the plant species were compiled on spreadsheets, following our previously described methodology.^[6c]^ Chemical structures currently available in PubChem^[8]^ were exported as SDF files, while structures unavailable in PubChem were sketched as MOL files using ChemDraw software (Prime version 16, courtesy Perkin Elmer). SMILES strings were generated using OpenBabel.^[9]^

### 2.2 Dataset Preparation

Data preparation was mainly done using PostgreSQL tables, as described in our previous publication.^[6c]^ Generally, compound and source species information were carefully and manually retrieved and double checked. For example, a Python API was used to retrieve individual molecules with appropriate PubChem Compound ID (CID) as 2D SDF files alongside compound names and synonyms if present in PubChem.^[8]^ Additionally, the 2D MOL files for molecules not found in PubChem or ChemSpider^[10]^ (based on searches using the name from literature and canonical SMILES) were manually sketched using ChemDraw (Prime version 16, Perkin Elmer) based on the published 2D structures as in the referenced literature source, while comparing with data available in Scifinder.^[11]^ Furthermore, all SMILES were canonicalized with OpenBabel.[^6c^, ^12^] During this process, a unique InChI (the identifier of molecular global uniqueness) was assigned to each unique canonical SMILES.^[12c]^ For each molecule of the EANPDB, both the *inchified* SMILES and the corresponding InChI are provided on our online platform.

### 2.3 PAINS Analysis of EANPDB Content

The presence of certain structural features referred to as pan‐assay interference compounds (PAINS) have been established to certain behaviours (such as metal chelation, redox cycling and protein reactivity). that could interfere in assay readouts all the way from target to cell without any common mechanism involved. The compounds of EANPDB were screened to estimate the proportion of molecules that are predicted to be PAINS. PAINS analysis was performed using PAINS1, PAINS2, and PAINS3 filters, as implemented in Schrödinger's Canvas program.^[13]^

### 2.4 Diversity Analysis using Principal Components

Searching for novel compounds from a different chemical space with significant biological importance is currently vital in the field of drug discovery. This could be one approach towards facing the challenges of drug resistance. It is believed that such molecules could act *via* a different mechanism.^[14]^ In order to evaluate in the chemical space occupancy of the different datasets, a PCA using the MOE package was performed.^[15]^ Several selected descriptors were computed and transformed linearly using PCA to obtain a new and smaller uncorrelated and normalized table of descriptors (mean=0 and variance=1).^[16]^ The descriptors for this purpose included the number of *donor/accptHB*, number of heavy atoms present, the number rotatable bonds, calculated molecular weight, predicted molar refractivity, the predicted total polar surface area and the computed octanol/water partition coefficient. Percentage variation based on counts as well as 2‐ and 3‐dimensional plots of PCA1, PCA2 and PCA3 (the best three principal components) were used to depict the diversity of molecules.

### 2.5 Scaffold Diversity Analysis

In the field of small‐molecule drug discovery, scaffold novelty/diversity is an important issue for complicated (hard to treat) ailments.^[17]^ Chemical scaffolds have diverse usages and play a key role in lead compound identification.^[18]^ In order to evaluate the scaffold diversity within the published NP datasets from Africa, scaffolds for the unique molecules found in the EANPDB were analyzed in comparison to those of the updated NANPDB using the Platform for Unified Molecular Analysis (PUMA) platform.^[19]^ This platform makes use of integrated metrics to characterize compound databases including the visualization of scaffold content, fingerprint diversity among others from the user input file (a comma‐separated value (.csv) file having three columns; SMILES, database names, and compound IDs). In this study, both the Cyclic System Recovery (CSR)^[20]^ and Scaled Shannon Entropy (SSE)^[21]^ were used to analyse the scaffold of molecules having at least a ring system while ignoring molecules with no ring. In this work, the cyclic systems were defined after the iterative removal of all side chains of the molecule.

### 2.6 Drug‐likeness and DMPK Prediction

Unique SMILES were used to generate the 3D models as well as the calculation of drug metabolism and pharmacokinetics (DMPK) properties of the collected molecules in our dataset using LigPrep and QikProp packages, respectively, of the Schrodinger software, as previously described.^[22]^ Some of the computed properties of interest included molecular weight (*mol_MW*), the number of H‐bonds that would be donated/accepted by the solute to water molecules in an aqueous solution (*donor/accptHB*), the number of non‐trivial (not CX3), non‐hindered (not alkene, amide, small ring) rotatable bonds (*#rotor*), the computed octanol/water partition coefficient (*QPlogP*_*o/w*_), predicted IC_50_ value for the blockage of HERG potassium ion (K^+^) channels (*QPlogHERG*), predicted brain/blood partition coefficient (*QPlogBB*), predicted skin permeability (*QPlogK_p_*), the number of likely metabolic reactions (*#metab*), prediction of binding to human serum albumin (*QPlogK_hsa_*), the number of violations of Lipinski's “rule of five” (*RuleOfFive*) and the number of violations of Jorgensen's rule of three (*RuleOfThree*).

### 2.7 Toxicity prediction

In a similar protocol.,^[6c]^ *in silico* prediction of the toxicity was carried out on the freely accessible online pkCSM web server (Cambridge University) for all the EANPDB molecules.^[23]^ The pkCSM platform provides a prediction of several parameters related to absorptions, distribution, metabolism, excretion and toxicity (ADMET), which includes ten toxicity endpoints as seen in Table 1.

**Table 1:** A summary of some toxicity endpoints predicted by the pk‐CSM server (http://biosig.unimelb.edu.au/pkcsm/).

Prediction	Endpoint (unit/presence)	Recommended range
AMES toxicity	Categorical (Yes/No)	No
Max. tolerated dose (human)	Numeric (log(mg/kg/day))	<=0.477
hERG I inhibitor*	Categorical (Yes/No)	No
hERG II inhibitor**	Categorical (Yes/No)	No
Oral Rat Acute Toxicity (LD50)	Numeric (mol/kg)	***
Oral Rat Chronic Toxicity (LOAEL)	Numeric (log) mg/kg_bw/day)	***
Hepatotoxicity	Categorical (Yes/No)	No
Skin Sensitisation	Categorical (Yes/No)	No
*T. pyriformis* toxicity	Numeric (log μg/L)	<=0.5
Minnow toxicity	Numeric (log mM)	>0.3

*hERG I inhibitors are predicted from a model using information from 368 compounds while. **hERG II inhibitors were predicted from a model using information from 806 compounds. The prediction will determine if a molecule is an hERG I or hERG II inhibitor. ***Interpreted relative to the bioactive concentration and treatment length.

### 2.8 Case Study: Substructure Searching

Post‐translational modification of histone proteins by enzymes such as histone deacetylases (HDACs, which catalyse the deacetylation of lysine residues on histone tails) participate in several physiological processes and are considered potential drug targets for various diseases.^[24]^ Human HDACs are represented in eighteen isoforms which are grouped as zinc‐dependent (Classes I, II and IV) or NAD^+^‐dependent (Class III).^[25]^ The zinc‐dependent HDACs comprise of the following isoforms; class I (HDAC1‐3, HDAC8), class II (IIa: HDAC4‐5, HDAC7, HDAC9 and IIb: HDAC6, HDAC10) and class IV (HDAC11). Resolved crystal structures show that the catalytic domain is conserved. Interest in targeting these HDAC isoforms have led to the market approval of several HDAC inhibitors and more are intensively being developed.[^24a^, ^24b^, ^24f^] However, because of the conserved nature of the binding pocket, most inhibitors lack selectivity between the various HDAC isoforms.[^24a^, ^24b^] Thus, there is a need to search for novel HDAC inhibitors that can achieve selectivity among the structurally similar HDAC isoforms.

A classical pharmacophore model for HDAC inhibitors as proposed by Jung et al.^[26]^ consists of three features; a zinc‐binding group (ZBG) coordinating the catalytic zinc ion, a linker placed in the hydrophobic substrate‐binding tunnel and the capping group (cap) that interacts with the rim of the pocket. An attempt to search for novel and selective HDAC inhibitors has been to search for a new ZBG that can make a significant contribution to the binding affinity. Amongst the reported HDAC inhibitors from nature are the macrocyclics; romidepsin and largazole, having a thiol ZBG in their activated forms.^[27]^ In this regard, substructure searching,^[24g]^ which represents a simple but powerful tool in drug discovery to perform initial filtration of molecules implemented in our online database was used to search for molecules with a thiol group, sulphur containing ZBG or sulphur‐containing molecules that can also be activated to HDAC inhibitors.

## 3 Results and Discussion

### 3.1 Overview of Data

This study provides a simple to use and interactive online platform containing data for isolated NPs from sources of East African origin. For the sake of preserving the knowledge of the traditional application of medicinal plants in this region of Africa, this comprehensive database was developed *via* the manual curation of literature sources. The entire content of this database (which includes: information about source species and country of harvest, isolated molecules, reported biolocigal activity, predicted drug‐likeness properties amongst others) can be accessed and downloaded from http://african‐compounds.org The current release of EANPDB is summarized in Table 2 below. This includes information from 315 citable literature sources, of which less than half of this material could be found in PubMed, with only 154 references having PubMed IDs (PMIDs).

**Table 2:** Summarized content of the EANPDB.

Number of references	315
Number of source organisms	302
Number of unique SMILES	1870
Number of compound classes	70
Number of unique PubMed IDs	154
Number of kingdoms	3
Number of unique PubChem IDs	1115
Number of biological activities	82
Number of families	58
Numbers of compounds identified for the first time	515
Molecules with reported biological activity	815

**Figure 1:**
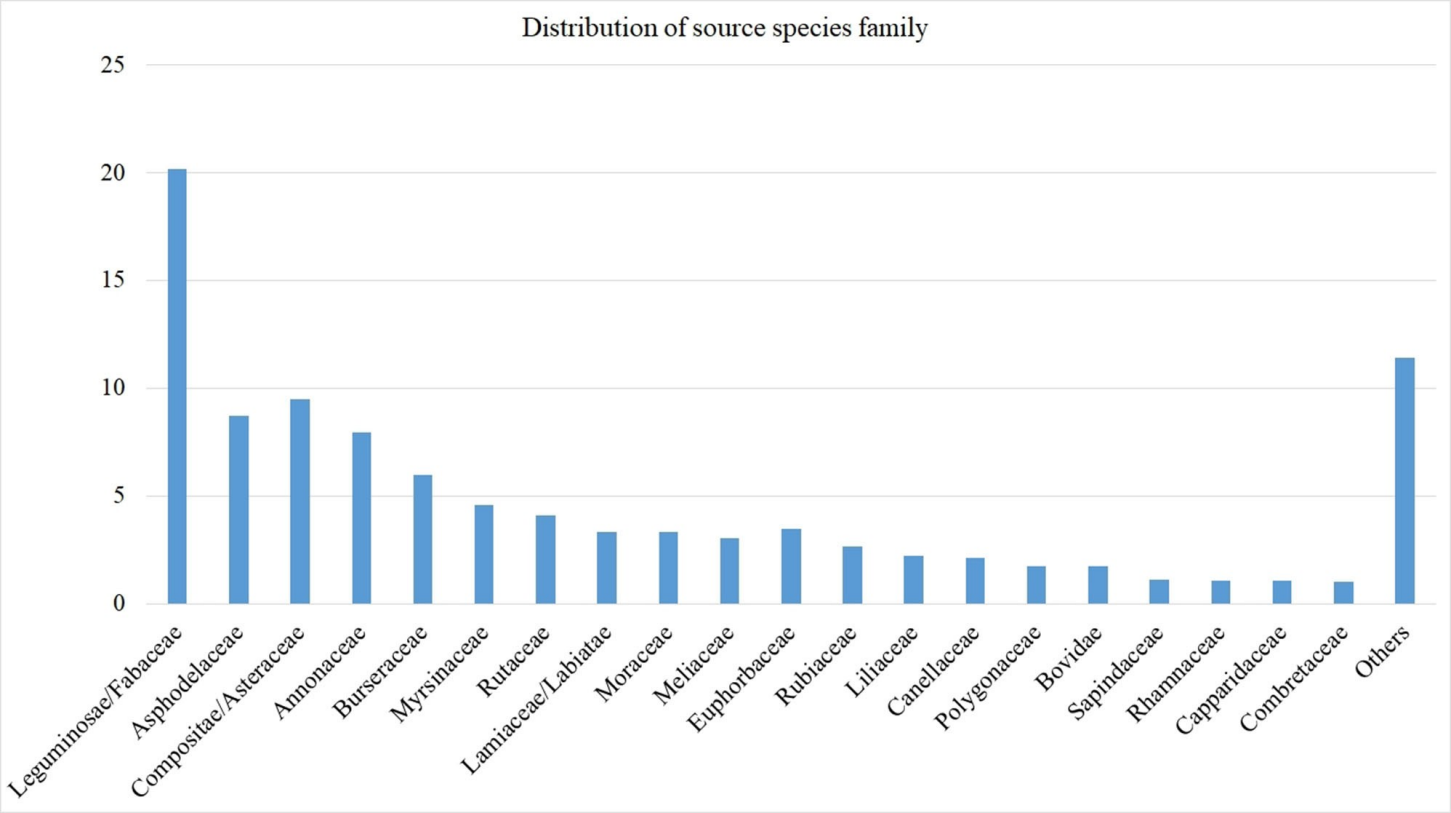
Bar chart showing the percentage contribution by family.

The data included 1,870 unique compounds from 302 source organisms from 3 kingdoms, belonging to 60 families (Figure 1). It was observed that the major contributing families occupying >50 % of the explored source species families were Leguminosae (Fabaceae) (∼20 %), Compositae (Asteraceae) (∼10 %), Asphodelaceae (∼9 %), Annonaceae (∼8 %) and Burseraceae (∼6%).

**Figure 2:**
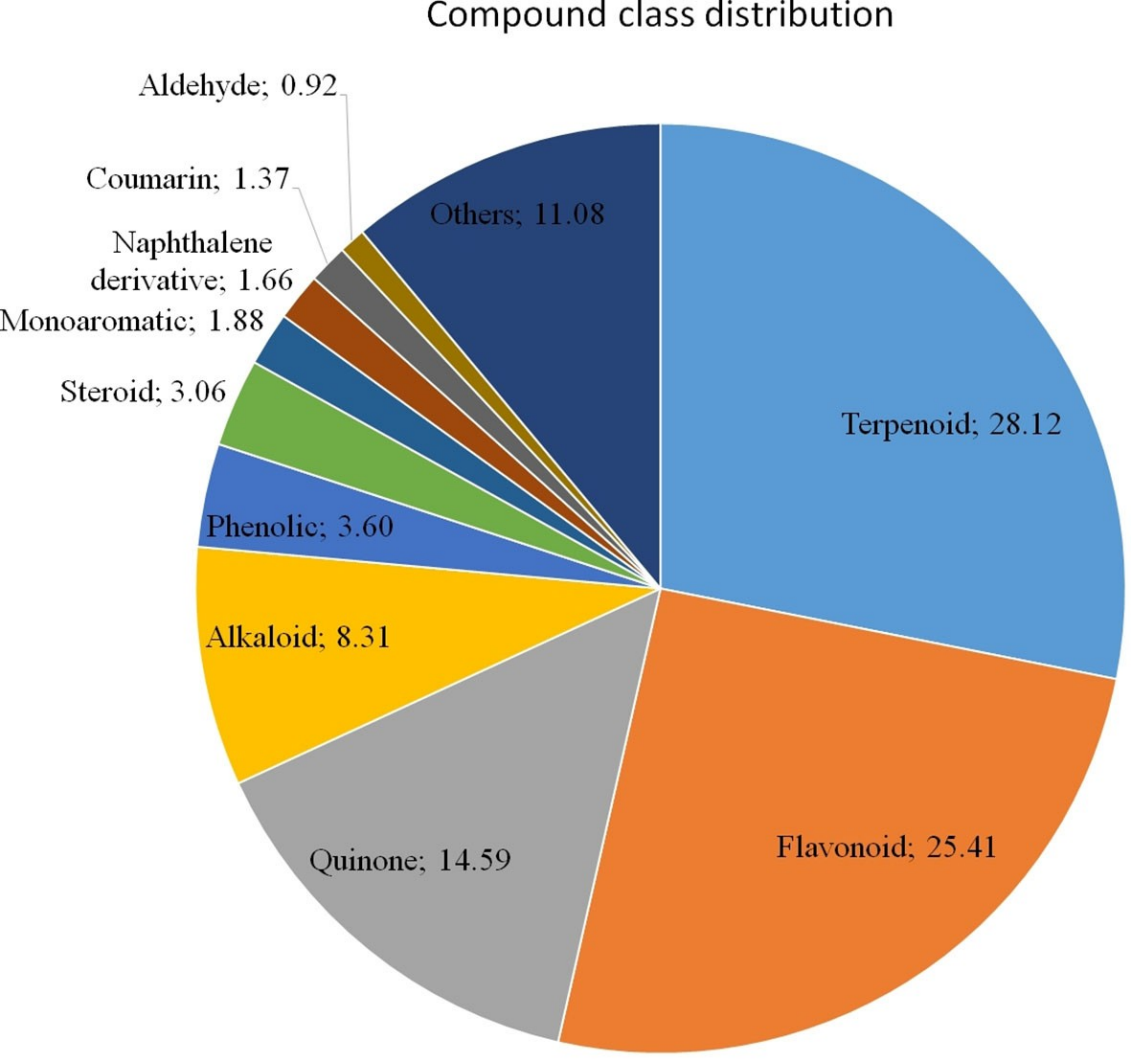
Distribution of main compound classes.

Approximately 40 % of the molecules had no PubChem ID (CID). Additionally, >500 of the molecules at the time of isolation and characterization were being reported for the first time. Giving the chance, several other new molecules can still be isolated from some of the less explored plant families (Figure 1). We observed a total of 70 compound classes, with the major contributors being terpenoids, flavonoids, quinones, alkaloids and phenolics (summing up to >80% of all the molecules currently in EANPDB, Figure 2).

**Figure 3:**
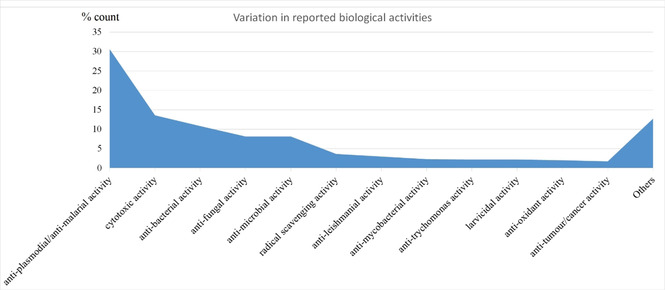
Distribution of reported biological activities within the EANPDB.

**Figure 4:**
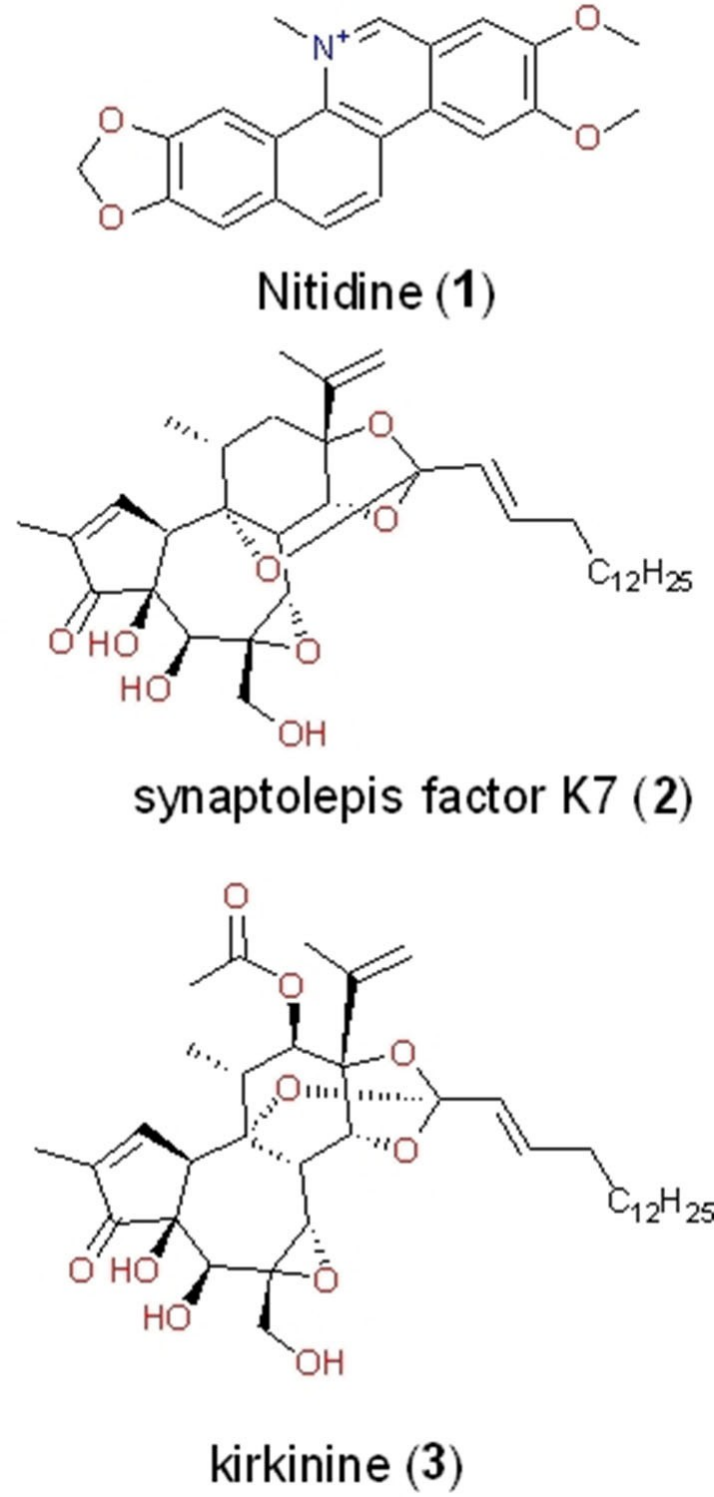
Molecules from the EANPDB with reported mechanisms of action (MOA).

Interestingly, this trend of the top compound class occupiers is very similar to what was observed in the Northern African NP database.^[6c]^ Additionally, about 44 % of the molecules in the EANPDB have at least one reported biological activity from a broad list of activities curated from the literature. These reported activities have been grouped into 40 classes. Anti‐malarial/anti‐plasmodial evaluations were the most reported (Figure 3). This is following the fact that malaria and other parasitic diseases remain serious burdens to the people in this region.^[28]^ Thus, the scientific validation of most of the traditional application of medicinal plants goes into confirming the usage of such plants in treating parasitic diseases such as plasmodial related ailments. Also, a few molecules from East African sources with interesting anti‐viral (HCV and HIV) activities were reported and are present in this version of the database. To further validate some of the reported activities, mechanism of action (MOA) for 3 of the molecules have been confirmed and has been curated in the EANPDB. These molecules include nitidine (**1**), synaptolepis factor K7 (**2**) and kirkinine (**3**) (Figure 4).[^29^, ^30^, ^31^]

Nitidine (**1**) was isolated by Rashid et al., from *Toddalia asiatica* (Rutaceae); a plant used traditionally in several communities in Kenya for the treatment of malaria and other ailments.^[29]^ Reports have it that all parts of the plant are claimed to have medicinal values, but the roots, in particular, are believed to be more potent.^[30]^ This plant is administered traditionally as decoction or infusion of the roots for patients to drink for the treatment of malaria, fever and to cure stomach ache. For toothache, the root is chewed whereas for the treatment of coughs the fruits are chewed. In the study, Rashid et al. evaluated and reported that compound **1** is an anti‐HIV‐1 molecule capable of inhibiting HIV‐1 reverse transcriptase. Although this molecule showed interesting anti‐HIV activity, it was also cytotoxic to the host cells at approximately 15 μg/ml.^[29]^ Thus, just like many other reported NPs, further optimization of nitidine (**1**) could serve as a good starting point in the development of novel and potent anti‐HIV drugs.[^1c^, ^1e^, ^2b^, ^2c^, ^2d^, ^6f^] Other molecules isolated from EA source species with reported modes of action include the daphnane diterpene esters; synaptolepis factor K7 (**2**) and kirkinine (**3**), both reported as having anti‐tumoral and neurotrophic actions, acting *via* the modulation of protein kinase C.^[31]^ These compounds were isolated from the roots of *Synaptolepis kirkii* (Thymelaeaceae), which is commonly used to manage epilepsy and snake bite.^[31]^ Although the evaluated activities and proposed modes of action of the compounds **2** and **3** deviated from the local traditional usage of this plant species, this buttresses the point that NPs from medicinal plants have a broad spectrum/range of activities and can be chemically modified to suit our target of interest.[^1b^, ^1c^, ^2b^, ^2c^, ^3a^, ^6d^]

In brief, this opens another corridor of scientific research on NPs from this area, which can be focused on the evaluation of the MOA of isolated NPs from this region, since very little has been done in this regard. Furthermore, PAINS investigation of the molecules present in EANPDB revealed that only 241 molecules (∼13 %) had scaffolds that could be predicted with an endpoint for the presence/manifestation as a PAINS. The list of matching PAINS and the number of scaffolds containing them are summarized in Table SI_1. In a similar way as previously reported by Baell,^[32]^ the majority of the molecules (∼95 %) from our dataset filtered as PAINS were molecules having the catechol(s) or quinone(s) scaffolds/fragments. Based on reports obtained from literature sources, it is confirmed that catechols and quinones can interfere in bioassays via different mechanisms such as metal chelation, redox cycling, redox activity as well as covalent reaction with biological targets and protein reactivity – which has been attributed to some observed toxic effects.^[33]^

Additionally, comparative studies between EANPDB with other compound databases (e.g. DrugBank,^[34]^ StreptomeDB,^[12a]^ NANPDB^[6c]^ and NuBBE^[5e]^) will be discussed.

In a nutshell, EANPDB was combined with NANPDB to form ANPDB and can be accessed via http://african‐compounds.org/. With ∼6500 unique molecules isolated from about 1000 source species, ANPDB represents the most extensive collection of African NPs available at the moment. The platform is built with an array of search fields that can be used on the entire African collection or narrowed down to specific regions, for example, the EANPDB. Some of the search methods include biological activity, compound name, source species, families and authors/reference. Similarity search and substructure search procedures are also implemented on our online platform. The structural similarity search makes use of the Tanimoto coefficient of similarity to measure the 2D similarity between the query molecule and the database entries. This tanimoto coefficient represents a number between 0 and 1; with 1 being the highest and referring to an exact match. The fingerprints used for the structural similarity search are pre‐calculated for all database entries and stored as blob objects in the PostgreSQL‐database. For each query structure, calculations are made during the search. We also provide users of our platform with an option to download the entire content as 2D or 3D SDF files or SMILES. Additionally, there is a help page to guide new users through our platform which also answers technical questions that might arise.

### 3.2 Chemical Space Analysis

**Figure 5:**
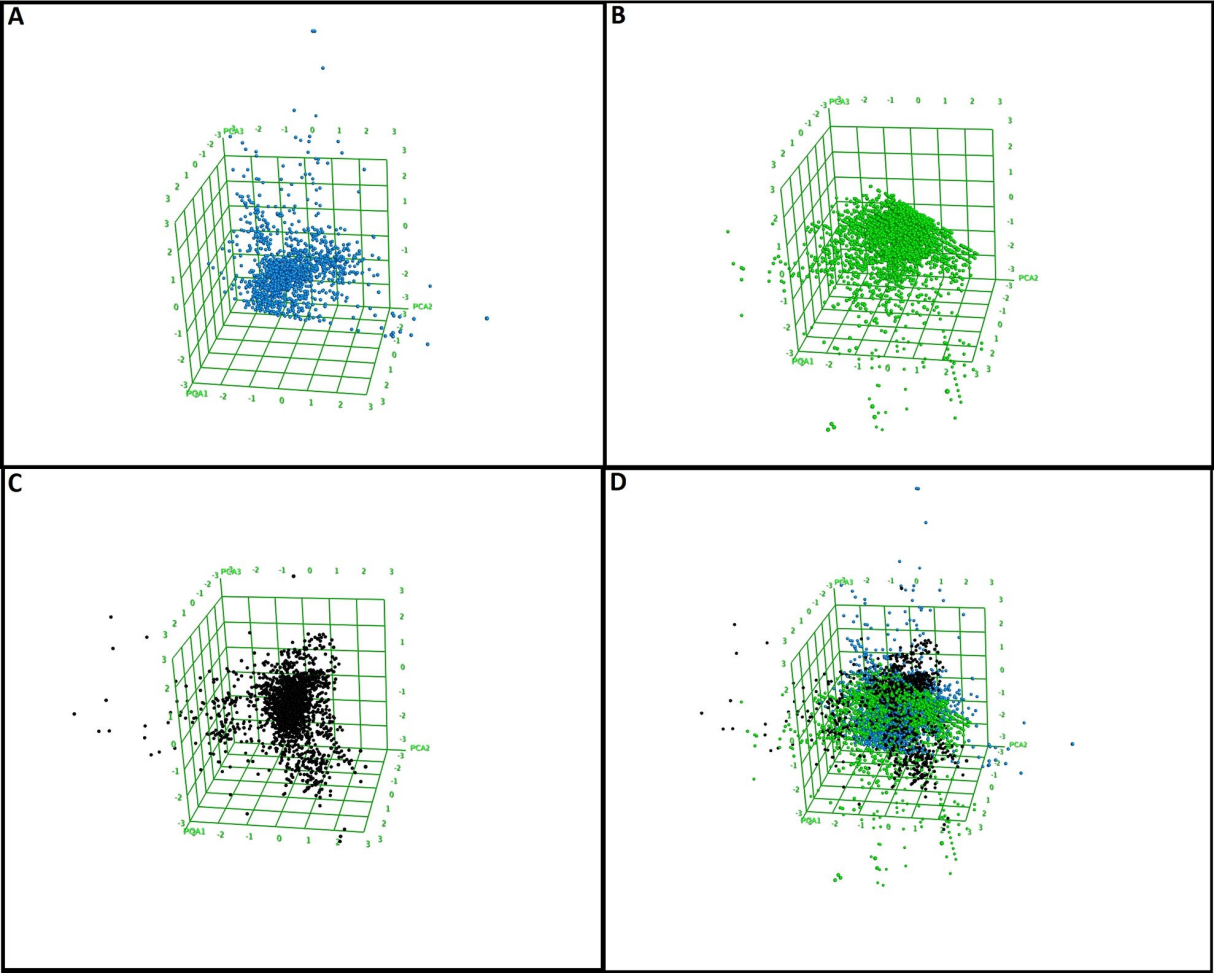
Chemical space analysis using the best 3 PCAs (PCA1, PCA2 and PCA3). A) Distribution of molecules in EANPDB, B) Distribution of molecules in NANPDB, C) Distribution of molecules in NuBBE and D) Overlay of all three datasets to appreciate their spatial distribution. Blue, Green and black balls represent EANPDB, NANPDB and NuBBE respectively.

A diverse dataset holds a key premise for the identification of novel molecules *via* screening methods when compared to a similar‐sized combinatorial library with limited structural variation.^[35]^ To analyze the chemical space coverage of the EANPDB, PCA in comparison with the updated versions of NANPDB^[6c]^ and NuBBE^[5e]^ was performed and the findings are presented here. The molecular diversity of the NPs constituting the EANPDB, using PCA is illustrated with the 3D scatter plots in Figure 5.

An analysis of the three most important principal components demonstrated that approximately 95 % covariation of the global information could explain the content of the various datasets, As observed in Figure 5, the three datasets clustered around at the centre, an indication of the molecules occupying similar chemical space. However, portions of each dataset tend to deviate outwardly in different directions. Thus, an indication of molecules that are chemically different from those represented at the centre. As observed, the top section of the scatter plots in the figures is highly represented by molecules from NuBBE, while the left part of the plot is occupied more by the EANPDB and the lower part of the figure shows an overwhelming presence of NANPDB molecules. Thus, the content of the EANPDB, NANPDB and NuBBE dataset molecules used in this study are observed to occupy diverse chemical spaces.

### 3.3 DMPK

**Figure 6:**
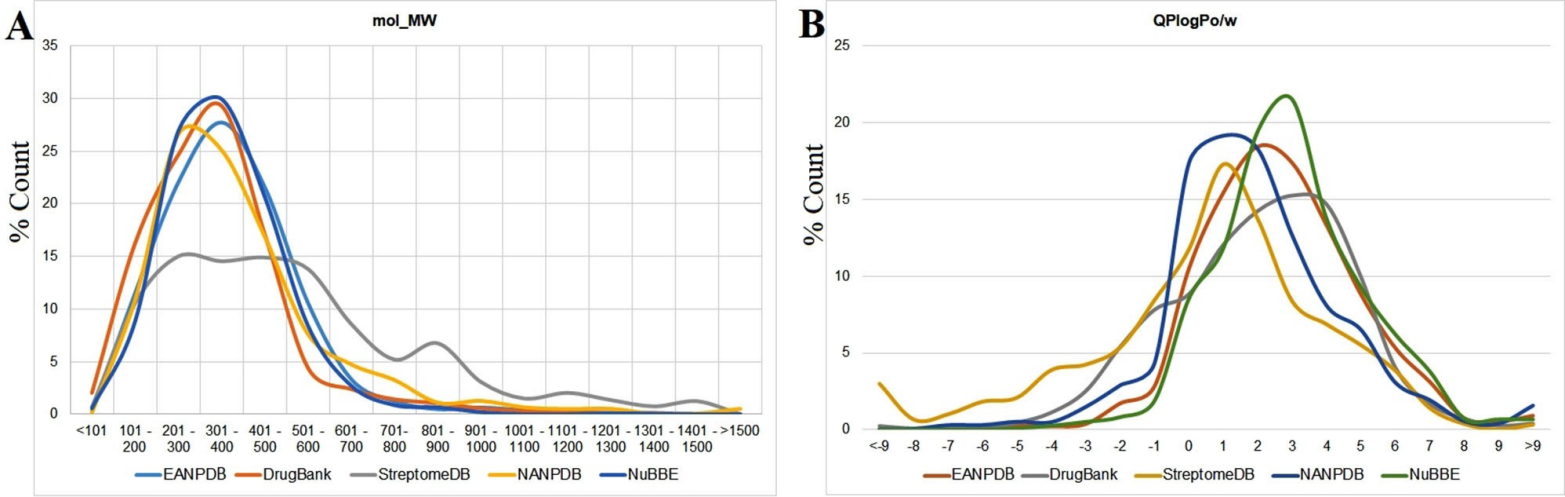
Distribution of A) molecular weight and B) logP (octanol‐water) coefficient for EANPDB, DrugBank, StreptomeDB, NANPDB and NuBBE datasets.

**Figure 7:**
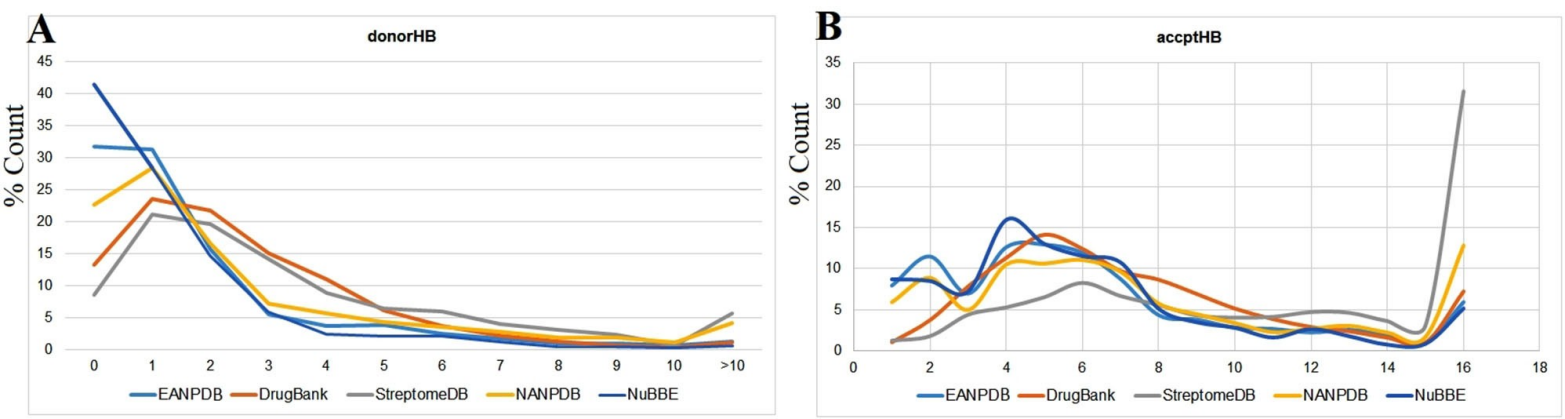
Distribution of A) H‐bond donor and B) H‐bond acceptor for EANPDB, DrugBank, StreptomeDB, NANPDB and NuBBE datasets.

**Figure 8:**
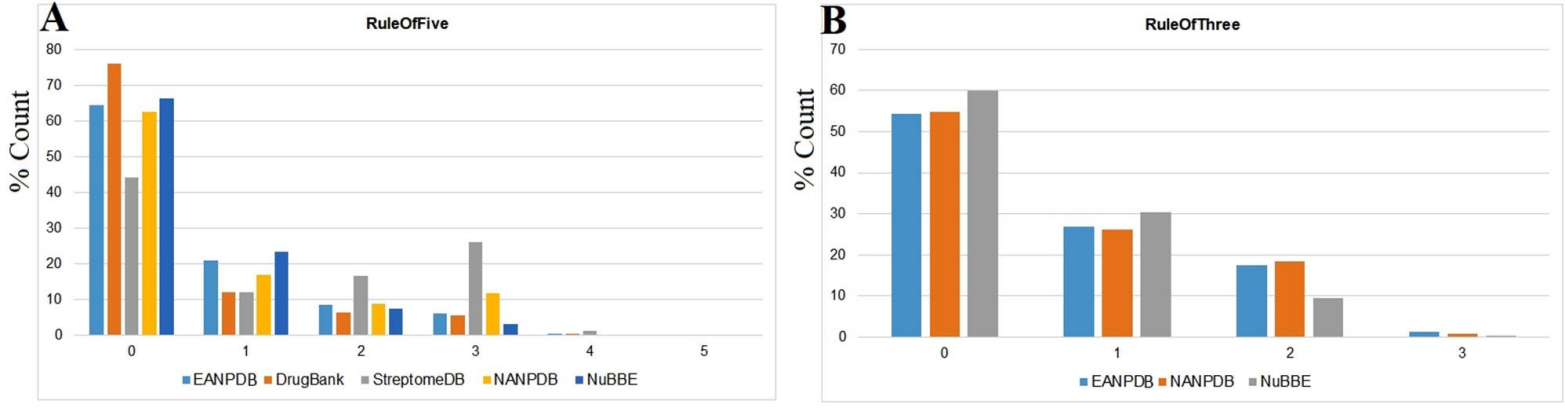
Percentile violation of A) drug‐likeness (Lipinski's rule of five violation) B) lead‐likeness (Jorgensen's Rule of Three violation).

A drug‐likeness study to show the distribution of the EANPDB molecules (Table 3) was further carried out using common druglikeness filters like molecular weight (*mol_MW*), logarithm of the octanol/water partition coefficient (*QPlogP*_*o/w*_), number of H‐bond donors (*donorHB*), number of H‐bond acceptors (*accptHB*), as well as an evaluation of the ratio of molecules violating the lead‐like rule of 3 (*RuleOfThree*) and the Lipinski rule of 5 (*RuleOfFive*) for orally available drug molecules. The variation in terms of percentages of these physicochemical properties among the selected datasets for comparison is shown in Figures 6, 7, 8.

**Table 3:** Some computed QikProp descriptors. The maximum (Max), minimum (Min) and average (Avg) values for molecules in the EANPDB as well as the recommended range.

Descriptors	Max	Min	Avg	Recommended range
*mol_MW* ^a^	1237.39	73.00	348.82	130.0–725.0
*QPlogP*_*o/w*_^b^	17.18	‐7.09	3.45	−2.0–6.5
*donorHB* ^c^	17.00	0.00	1.33	0.0–6.0
*accptHB* ^d^	44.50	0.00	5.04	2.0–20.0
*#rotor* ^e^	47.00	0.00	6.04	0–15
*QPlogBB* ^f^	2.43	−10.13	−1.05	−3.0–1.2
*QPlogK_p_* ^g^	10.13	−14.35	−2.71	−8.0–−1.0
*QPlogHERG* ^h^	‐0.83	−8.47	−4.59	concern below −5
*#metab* ^i^	0.00	20.00	4.53	1–8
*TPSA* ^j^	1621.47	244.81	619.67	300.0–1000.0
*QPlogS* ^k^	0.98	−18.87	−4.62	−6.5–0.5
*QPlogK_hsa_* ^l^	4.11	−3.15	0.33	−1.5–1.5

^**a**^ Molecular weight (DA), ^**b**^ Logarithm of the octanol/water partition coefficient, ^**c**^ Number of H‐bond donors, ^**d**^ Number of H‐bond acceptors, ^**e**^ Number of rotatable single bonds, ^**f**^ Logarithm of the blood/brain barrier partition coefficient, ^**g**^ Logarithm of the skin permeability coefficient, ^**h**^ Logarithm of the HERG blockage coefficient, ^**i**^ Number of metabolites, ^**j**^ Total polar surface area, ^**k**^ Logarithm of the water solubility parameter, ^**l**^ Logarithm of the binding constant to human serum albumin.

As observed in Figure 6A, the data indicate that the distribution of *mol_MW* generally followed a Gaussian‐like curve with maximum peaks around 300 Da. While molecules with *mol_MW* <500 Da dominated the EANPDB, NANPDB, DrugBank and NuBBE datasets, the StreptomeDB dataset rather demonstrated an almost level distribution in the number of molecules with ∼200 Da<*mol_MW*<∼550 Da. Approximately 85 % of the molecules contained in all the datasets analyzed had MW less than 500, thus meeting one of the Rules of Five for oral drugs. Similarly, the distribution of molecules concerning the predicted *QPlogP*_*o/w*_ below 5 also dominated (∼85 %). All calculated *QPlogP*_*o/w*_ for molecules in the different datasets used in this study for comparison followed a similar distribution pattern (Figure 6B).

Furthermore, analysis of the count of donor/accptHB (Figure 7) content for each of the utilized datasets showed that about 80 % of the molecules in each dataset had respected the conditions of *donor/accptHB* in the rule of 5. From the obtained *donor/accptHB* data, we could observe a similar distribution for the EANPDB, NANPDB and DrugBank. It was remarkable that approximately 42 % of molecules in the NuBBE dataset had no *donorHB* (Figure 7A) while the StreptomeDB dataset, on the other hand, had about 40 % of its molecules fulfilling the required criterion for the accptHB filter (Figure 7B). However, this difference in the observed distribution of *donor/accptHB* stands to confirm the fact that the composition and diversity of molecules from StreptomeDB (containing NPs from *Streptomyces sp*.) has not been studied systematically and quantitatively and may vary otherwise from NPs from plant sources.

An evaluation of the computed physicochemical properties of the violation of the “Rule of Five” is summarized in Figure 8. The molecular enumeration showed that a majority of molecules in the EANPDB (∼65 %) had no Lipinski's (Rule of Five) violation, while approximately 85 % of all the molecules in the analyzed datasets had <= 2 Lipinski's violation (Figure 8A). Additional investigation of how lead‐like the content of the three NPs datasets (EANPDB, NANPDB and NuBBE; mainly from plant sources) was performed (Figure 8B). It was observed that about 55 % of the NPs from the Northern and Eastern parts of Africa had no lead‐like (Rule of Three) violation while ∼60 % of the Brazilian collection (NuBBE) had no lead‐like violation.

### 3.4 Toxicity Prediction

The application of *in silico* methods such as virtual screening and toxicity prediction have continuously gained grounds to complement resource expensive wet laboratory experiments in the drug discovery pipeline. This is especially the case when it comes to NPs that are normally available only in low yields. A good proportion of the molecules were predicted as negative (complied) with the AMES mutagenic test in bacteria (Table 3). A negative prediction with the AMES text indicates that the compound in question is not mutagenic and may not act as a carcinogen. On the other hand, almost all the molecules in EANPDB (99.7 %) were predicted as not interfering with the inhibition of the potassium ion (K^+^) channels (encoded by hERG I). About 85 % of the content of the EANPDB were predicted to have no hepatotoxic or skin sensitization effect. The human maximum tolerated dose (Max. tolerated dose in log mg/kg/day) extrapolated from animal data that gives an idea of the maximum recommended starting dose in phase I clinical trials (the toxic dose threshold of chemicals in humans) has been predicted for each molecule. The maximum (Max), minimum (Min) and Average (Avg) of the *Max. tolerated dose* (human) alongside the Oral Rat Acute Toxicity (LD_50_), *Oral Rat Chronic Toxicity* (LOAEL), *Tetrahymena pyriformis toxicity* and *Minnow toxicity* have been summarized in Table 4.

**Table 4:** Some of the toxicity prediction endpoints. The maximum (Max), minimum (Min) and average (Avg) values for molecules in the EANPDB, along with the recommended range.

Toxicity endpoints	Max	Min	Avg	Recommended range
*Max. tolerated dose* (human)	2.35	−2.97	0.17	<=0.477
*Oral Rat Acute Toxicity* (LD_50_)	4.78	1.10	2.38	***
*Oral Rat Chronic Toxicity* (LOAEL)	11.94	−0.80	2.02	***
*Tetrahymena pyriformis toxicity*	2.59	−1.86	0.49	<=0.5
*Minnow toxicity*	22.99	−11.28	0.98	>0.3

*** Interpreted relative to the bioactive concentration and treatment length.

### 3.5 Scaffold Analysis

**Figure 9:**
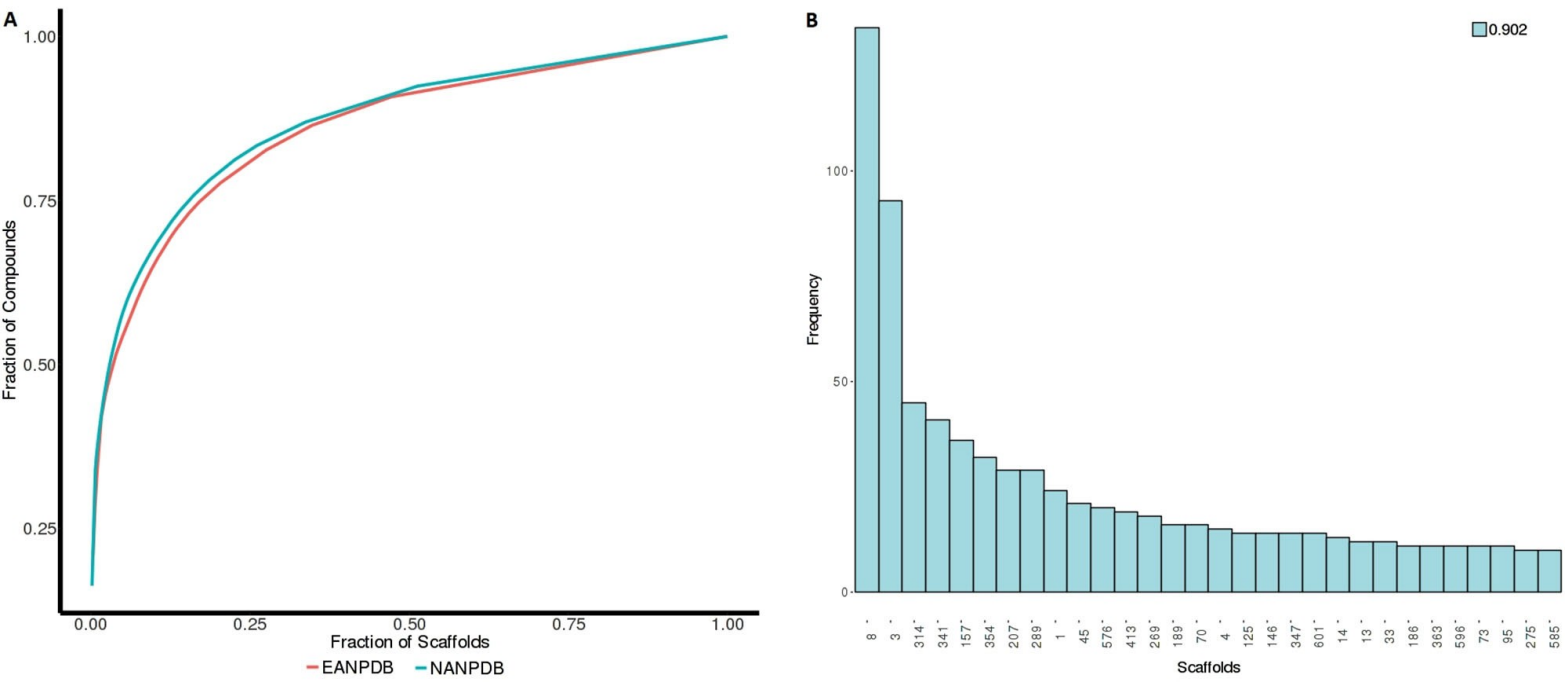
Distribution of scaffold similarity between EANPDB and NANPDB using A) CSR and B) SSE30.

**Figure 10:**
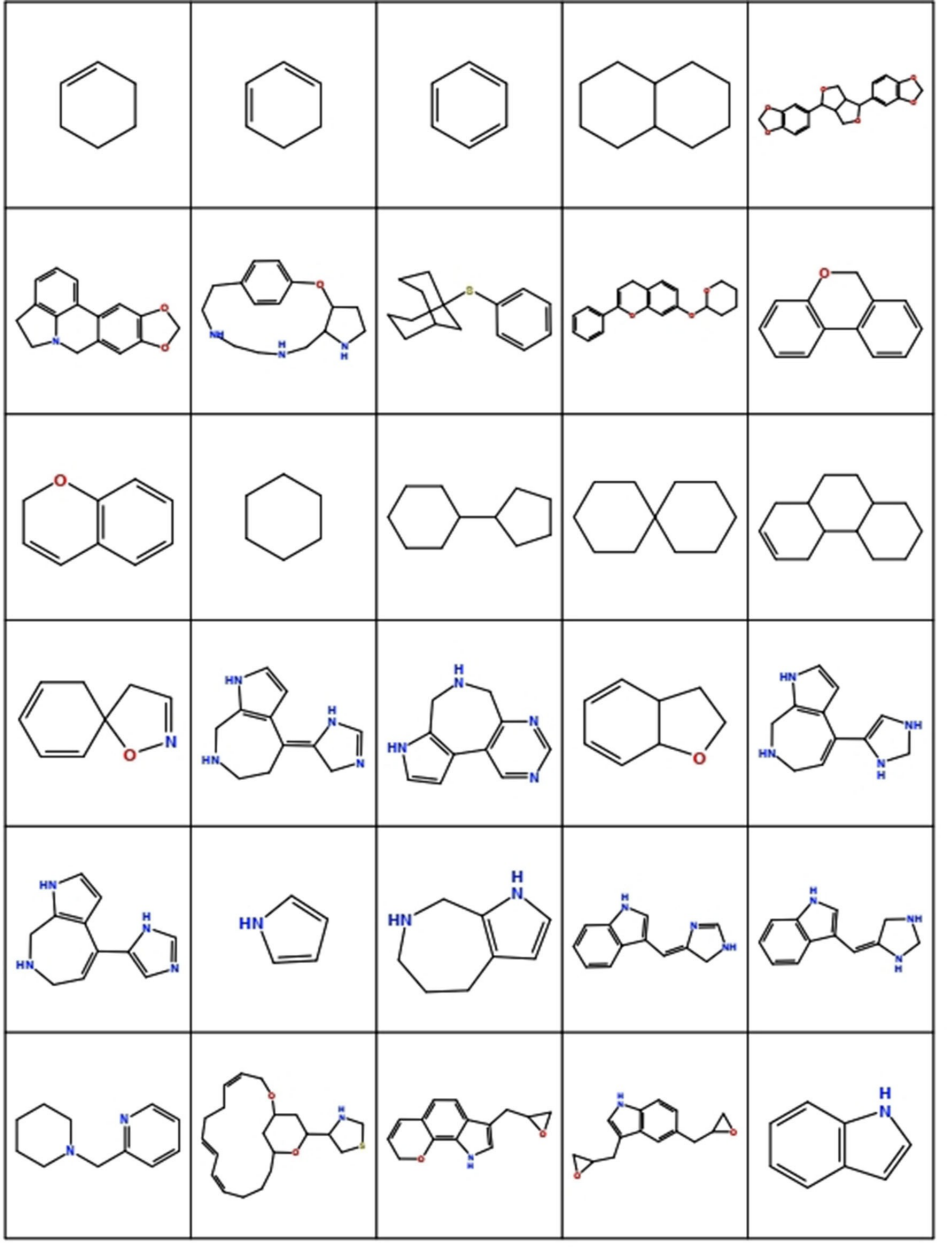
Display of most frequent cyclic scaffolds.

Molecules with ring system(s) were used in this analysis while those that do not have any rings were ignored. However, we observed that molecules without a ring system represented a very small minority of the complete datasets and therefore should not bias the overall scaffold analysis. The output file downloaded from the Scaffold (CSR curves) tap in PUMA shows that there is little scaffold diversity within the NANPDB and EANPDB (Figure 9). A highly diverse dataset is considered to have a CSR area under the curve (AUC) of about 0.5; an indication that there is almost one scaffold for each compound. The downloaded summary statistics (Tables S1; downloaded from “Download unique scaffolds”) confirmed that most of the compounds are cyclic (Figure 10). Both datasets have similar diversity in terms of scaffolds with AUCs of 0.86 and 0.87 and SSE30 of 0.90 and 0.91 for EANPDB and NANPDB respectively (Tables SI_2–4 and Figures SI_1, 2).

### 3.6 Identification of Latrunculins A and B from the Online African NP Database using a Substructure Search

**Figure 11:**
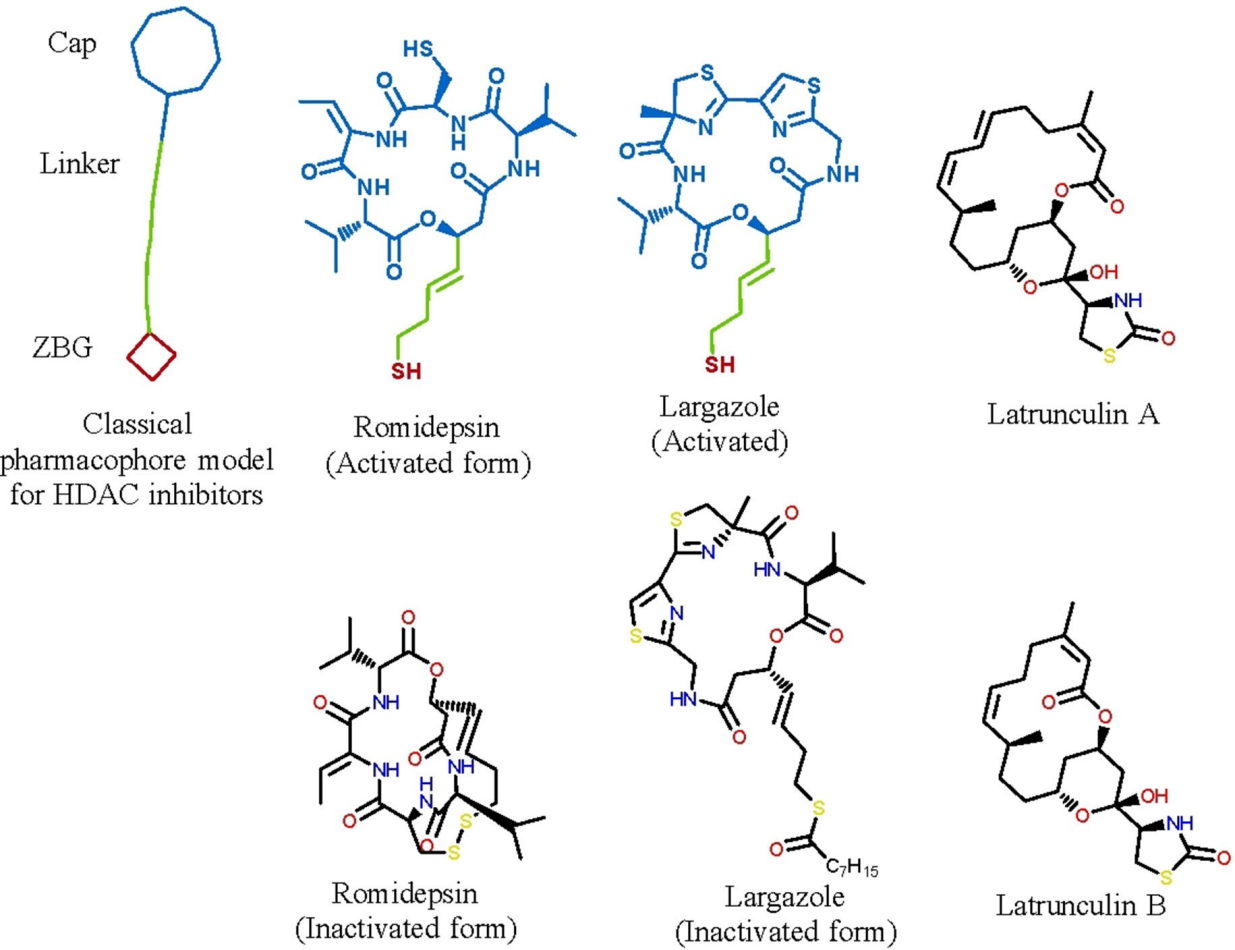
Classical pharmacophore of HDAC inhibitors and the Chemical structure of romidepsin, largazole and the newly proposed latrunculins A and B from the African NP database.

In order to evaluate the application of the African NP database, we applied a substructure search for molecules having a methylthiol moiety (chemical formula; CH_3_SH and canonical SMILES; CS). The existence of several thiol based HDAC inhibitors such as romidepsin and largazole (both prodrugs that generate the thiol *in vivo)* supports our idea to find molecules that possess sulphur containing functional groups from our database that can be used as a ZBG in the development of HDAC inhibitors (Figure 11).

**Figure 12:**
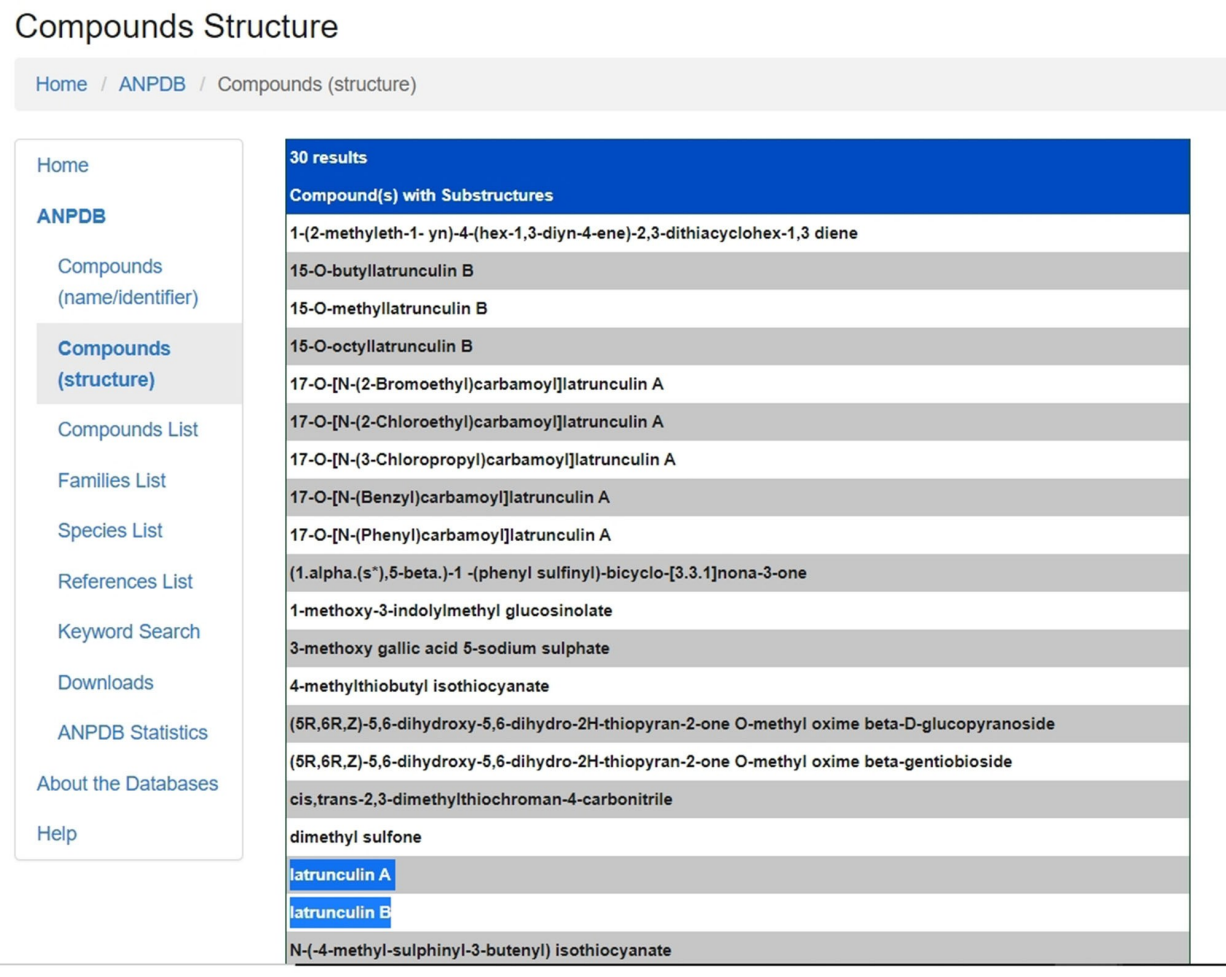
Partial “CS” substructure search output result.

A substructure search using CH_3_SH yielded 30 different molecules (Figure 12), mostly derivatives of latrunculins A and B (Figure 11). These molecules are mostly extracted from *Negombata magnifica* (Podospongiidae).^[36]^ The previously reported biological activity is antitumour activity, with mode of action proposed as binding reversibly to actin monomers, forming a 1 : 1 complex with G‐actin and disrupting its polymerization. Interestingly, latrunculins A and B both had no Lipinski's violation and their toxicity prediction showed that they can be good clinical candidates. It was interesting to observe that these molecules possess antitumour activity and possess a thiazolidin‐2‐on group that might act as a ZBG or may be activated to function as HDAC inhibitors. Moreover, HDACs have been reported to be new and interesting drug targets in the search of novel cancer/tumour related drugs.[^24e^, ^37^] It is left to question whether these molecules are inhibitors of HDACs as another unexplored or not reported mode of action.

## 4 Conclusions

NPs, especially those from African sources deserve more attention as they have been proven to be underrepresented, unexplored and understudied for drug discovery. NPs have been reported to contribute strategically in the drug discovery process either as new drug molecules or the relevant scaffold for the synthesis of novel bioactive molecules. In this study, we are continuing our effort to provide an online free database of NPs from African sources.[^6b^, ^6c^, ^6d^, ^6e^, ^6f^, ^6g^] The current collection herein represents the most comprehensive collection of NPs from the Eastern Africa region covering the period 1962 to 2019. This collection contains relevant details such as possible modes of action, as well as predicted toxicity, compound SMILES, 3D models computed physico‐chemical properties to estimate pharmacokinetics and pharmacodynamic properties, and literature reference of source data. It was shown that a significant portion of the molecules is not annotated in the PubChem database as well as the relatively few references found in PubMed. Additionally, just like the well‐known NP databases, comparative studies indicated that the EANPDB can be a good starting point for virtual screening based on the DMPK and Toxicity predictions made. Substructure searching starting from the methylthiol group (as a case study) for the identification of new HDAC inhibitors yielded molecules that were mostly latrunculins A and B derivatives. Latrunculins A and B have been reported to possess antitumour activity and *in silico* evaluation of these molecules show typical characteristics of good drug candidates.

## Supporting Information Available

Details on EANPDB content, molecular structures and their sources of the collection as well as computed descriptors can be accessed free of charge via the Internet at http://african‐compounds.org. The metadata is also available as additional spreadsheets.

## Conflict of interest

None declared.

## Supporting information

As a service to our authors and readers, this journal provides supporting information supplied by the authors. Such materials are peer reviewed and may be re‐organized for online delivery, but are not copy‐edited or typeset. Technical support issues arising from supporting information (other than missing files) should be addressed to the authors.

Supplementary

Supplementary

Supplementary

Supplementary

Supplementary

Supplementary
