# Supplementary figures and images for: Pharmacoinformatic Investigation of Medicinal Plants from East Africa
Source: Mol Inform. 2020 Oct 8;39(11):2000163. doi: 10.1002/minf.202000163 (PMC7685152; doi:10.1002/minf.202000163)

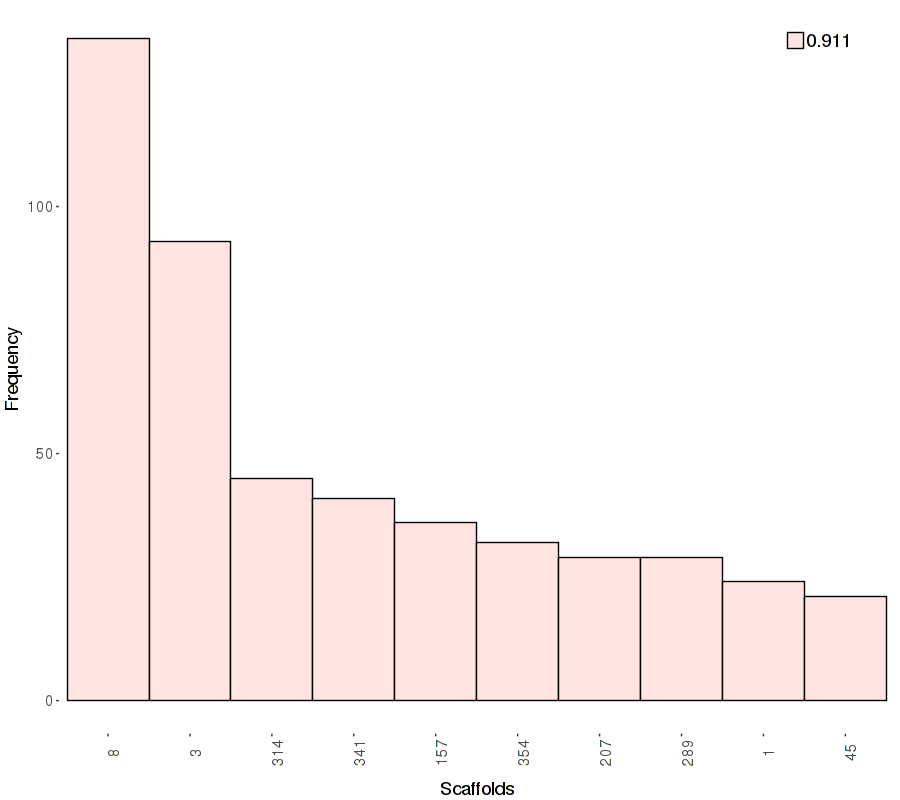

Supplement: Supplementary file 1 — Supplementary [file MINF-39-2000163-s001.tiff]

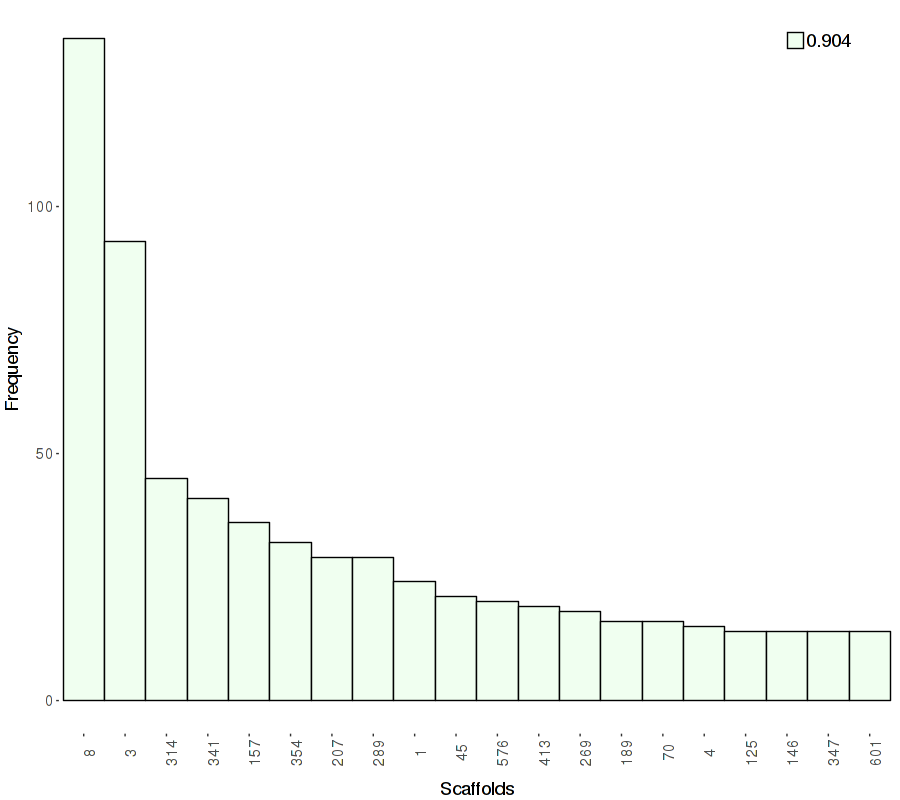

Supplement: Supplementary file 2 — Supplementary [file MINF-39-2000163-s002.tiff]
